# From open to robotic surgery in pediatric ureteral reimplantation: overcoming the learning curve for improved outcomes

**DOI:** 10.3389/fsurg.2025.1573233

**Published:** 2025-04-29

**Authors:** G. Mattioli, F. Fanti, M. Carlucci, S. Parodi, V. Fiorenza

**Affiliations:** ^1^ Department of Neuroscience, Rehabilitation, Ophthalmology, Genetics, and Maternal and Child Science, University of Genoa, DINOGMI, Genoa, Italy; ^2^ Pediatric Surgery Unit, IRCCS Istituto Giannina Gaslini, Genoa, Italy; ^3^ Epidemiology and Biostatistics Unit, Scientific Directorate, IRCCS Istituto Giannina Gaslini, Genoa, Italy

**Keywords:** robotic surgery, ureteral reimplantation, vesicoureteral reflux, megaureter, learning curve

## Abstract

**Introduction:**

With growing role of minimally invasive surgery, laparoscopic ureteral reimplantation (LUR) and robotic ureteral reimplantation (RALUR) have gained popularity in pediatric vesicoureteral reflux (VUR) treatment. However, literature on complex ureterovesical junction (UVJ) pathologies remains limited. This study reports RALUR outcomes over time and compares them with those of LUR and open ureteral reimplantation (OUR) for UVJ pathologies at a tertiary center.

**Materials and methods:**

Data from 80 RALUR (18 non dismembered ND-RALUR and 62 dismembered D-RALUR) performed in 2018–2023 were prospectively collected and compared with data from 21 LUR (16 non dismembered ND-LUR and 5 dismembered D-LUR) in 2018–2020 and 61 OUR in 2014–2021. Surgical indications included VUR, obstructive megaureter (OM), and refluxing obstructive megaureter (ROM), even in duplex system, bladder diverticula, ureterocele and prior UVJ surgeries.

**Results:**

Median age and weight were 2.1 years, 12 kg (OUR), 1.7 years, 13.5 kg (LUR), and 3.3 years, 15.0 kg (RALUR). No intraoperative complications occurred. Abdominal drainage and bladder catheterization were more frequent and lasted longer in OUR than in RALUR (*p* < 0.001). RALUR was associated with shorter hospitalization and reduced analgesic use (*p* < 0.001). Success rates were 79% (OUR), 50% (LUR), and 65% (RALUR), improving to 97%, 95%, and 98% after reinterventions. RALUR success increased from 55% (2020–2021) to 81% (2022–2023) (*p* = 0.02).

**Discussion:**

RALUR achieved comparable success to OUR and LUR while offering improved ergonomics, useful for complex cases, shorter hospital stays, and easier future endoscopic approaches. The increasing success rate reflects the robotic learning curve and growing surgical expertise.

## 1 Introduction

In pediatric urology, robotic surgery is particularly well-suited for procedures such as pyeloplasty and ureteral reimplantation, which require precise dissection and meticulous reconstruction. These technical demands make them ideal candidates for a robotic approach.

Robotic pyeloplasty is the most commonly performed robotic procedure in pediatric urology, with success rates exceeding 95%, comparable to those achieved with open surgery (1). However, the situation differs when addressing distal ureteral pathology. Regarding endourological treatments, recent literature reports resolution rates for high-pressure balloon dilation (HPBD) of 85%–89% for obstructive megaureter (OM), ranging from 64%–100%. However, the European Association of Urology (EAU) guidelines still do not recommend its routine use due to uncertainty regarding long-term outcomes. Similarly, endoscopic treatment for vesicoureteral reflux (VUR) achieves a 90% resolution rate, with reported success rates ranging from 50%–93%, which are not negligible (2–4). However, when endourological treatment is either not indicated or ineffective, ureteral reimplantation is required (4). Laparoscopic extravesical ureteral reimplantation (LUR) for primary vesicoureteral reflux (VUR) is a well-documented technique (5–7). Nevertheless, the technical complexity of ureteral mobilization, detrusor tunnel creation, and suture closure has led to a steeper and more challenging learning curve. Consequently, unlike minimally invasive pyeloplasty, LUR has not consistently yielded favorable outcomes, and open ureteral reimplantation (OUR) remains the gold standard for treating conditions such as VUR, OM, and refluxing obstructive megaureter (ROM) (1, 8, 9).

The adoption of robotic surgery represents a crucial step in transitioning toward minimally invasive management of these conditions. Robotic technology enhances precision in key surgical steps, including ureteral mobilization, detrusotomy, ureterovesical anastomosis, and detrusor muscle suturing, thereby optimizing the procedure (10). Robotic-assisted laparoscopic ureteral reimplantation (RALUR) has proven to be a safe and feasible approach for treating primary VUR, achieving success rates comparable to those of open surgery (11). However, its role in managing more complex ureterovesical junction (UVJ) pathologies—such as OM, ROM, complex duplex systems (DS), and paraureteral diverticula—remains to be clearly defined. To date, only case reports and small case series have been published in the literature (11–15).

This study aims to report RALUR outcomes over the years for the treatment of various UVJ pathologies at a tertiary center and to compare its results with those of LUR and OUR performed at the same institution.

## 2 Materials and methods

The study was approved by the Regional Ethics Committee of Genoa on July 23, 2021, under Protocol RR2020 No. 567/2020. Informed consent was obtained from the parents of all participants included in the study. Data from patients who underwent RALUR at our Institution between October 2018 and December 2023 were prospectively collected and analyzed. These data were then compared with those from a retrospective cohort of patients who underwent LUR and OUR at the same center between October 2018 and November 2020 and between February 2014 and November 2021, respectively. Additionally, the RALUR cohort was divided into two subgroups (RALUR 2020–2021 and RALUR 2022–2023) to analyze and compare success and reintervention rates over these two-year periods. All RALUR and LUR procedures were performed by a single surgeon, while OUR procedures were performed by multiple surgeons from the same institution. Inclusion criteria included OM, ROM, and grade IV–V VUR, even in cases of complex anatomy such as duplex systems, bladder diverticula, ureterocele, or prior surgical UVJ treatment. Additionally, grade II–III VUR was included if it was refractory to endoscopic treatment or present in patients with recurrent urinary tract infections (UTIs) and/or reflux nephropathy associated with complex anatomy or prior surgical UVJ treatment.

The exclusion criteria included: age ≥18 years, and follow-up of less than six months.

Collected data included demographic information, preoperative clinical and radiological findings, surgical details, postoperative outcomes, and clinical and radiological findings at follow-up.

Radiological workup included: abdominal ultrasonography (US) to assess the anteroposterior pelvic diameter (APD) and ureteral dilation, with evaluation for DS, ureterocele, and bladder diverticula, voiding cystourethrogram (VCUG) to confirm the presence of VUR, functional imaging as functional magnetic resonance urography (fMRU) or renal scintigraphy (Tc-99m MAG3 or DMSA) to assess UVJ obstruction and determine differential renal function (DRF). Preoperative assessments also included blood and urine tests.

For LUR and RALUR, patients with primary VUR without radiological signs of obstruction underwent non-dismembered reimplantation (ND-LUR or ND-RALUR), while those with secondary or persistent VUR after a previous ureteral reimplantation, OM, ROM, and periureteral diverticula, underwent dismembered reimplantation (D-LUR or D-RALUR). All patients in the OUR cohort were treated with Cohen cross-trigonal reimplantation.

Success was defined as the absence of symptoms (febrile UTI, flank pain) and improvement in pelvic and ureteral dilation on follow-up US. Febrile UTIs (fUTIs) were diagnosed based on fever (≥38°C), pyuria (urinalysis with positive leukocyte esterase), and single-organism bacteriuria (>100,000 CFU/ml).

Follow-up lasted a minimum of six months and included US at one, three, six, and twelve months postoperatively. VCUG was performed in cases of two or more fUTI episodes (with or without worsening dilation on US) to assess persistent or secondary VUR. Functional studies were conducted in cases of suspected postoperative UVJ obstruction or to evaluate DRF stability and potential improvement.

### 2.1 Statistical analysis

Continuous variables were presented as median and interquartile range (IQR), while categorical variables were expressed as absolute and relative frequencies. Due to the non-normal distribution of the continuous variables, group comparisons were performed using the non-parametric Mann–Whitney *U* test and the Kruskal–Wallis test for comparisons involving more than two groups. Associations between categorical variables were assessed using the chi-squared test or Fisher's exact test, as appropriate. A *p*-value < 0.05 was considered statistically significant. Bonferroni correction was applied to account for multiple testing bias.

### 2.2 Surgical technique

#### 2.2.1 OUR

OUR was performed using the Cohen technique (16). In cases of significantly dilated ureters, ureteral tailoring, as described by Starr (17) or Kalicinski (18), or tapering, as described by Hendren (19), was performed.

#### 2.2.2 LUR and RALUR

**Figure 1:**
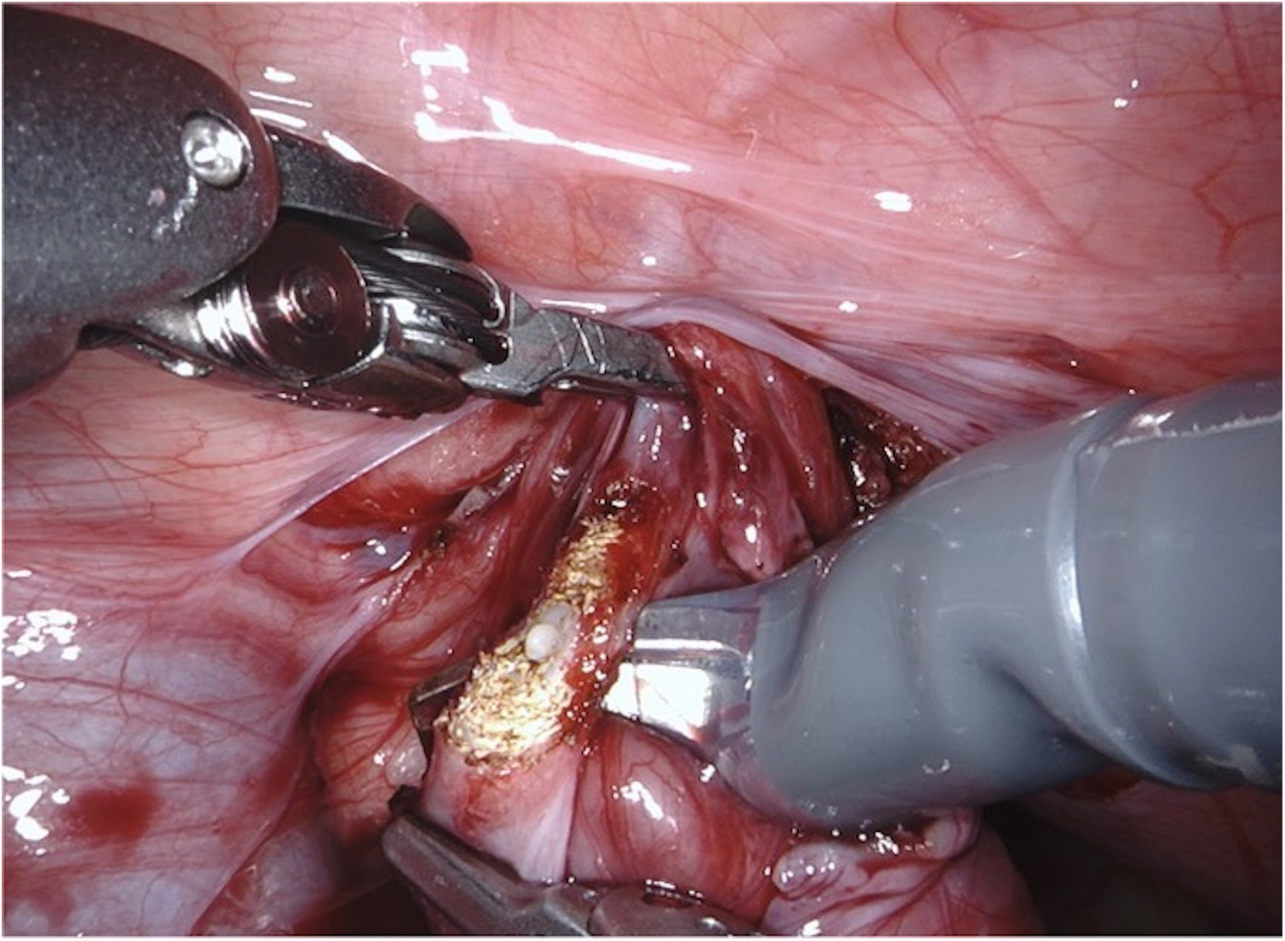
D-RALUR for OM. The distal ureter is identified and excised from the UVJ as distally as possible, and the terminal part is removed.

**Figure 2:**
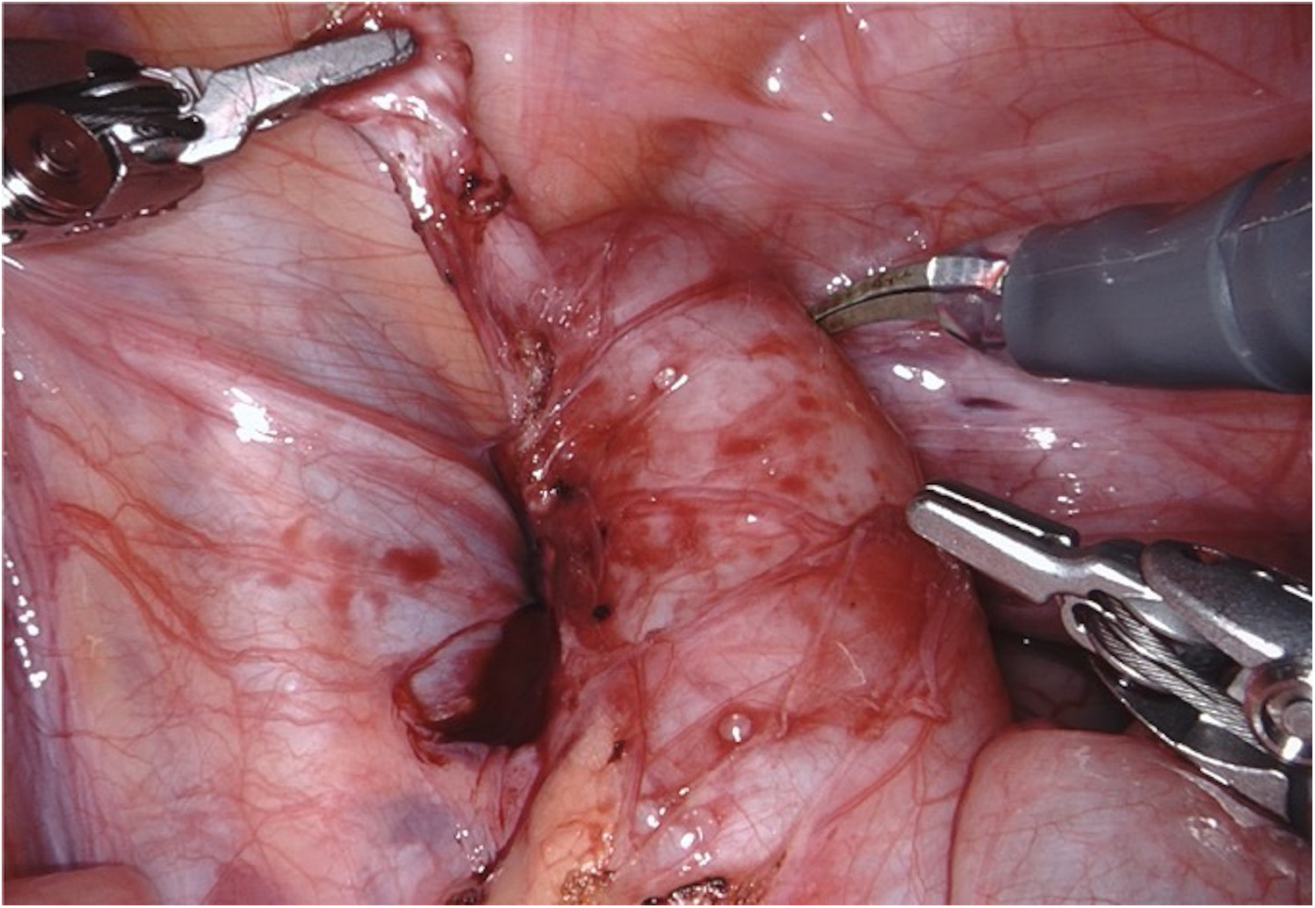
D-RALUR for OM. The “rat-tail” configuration of the distal ureter.

**Figure 3:**
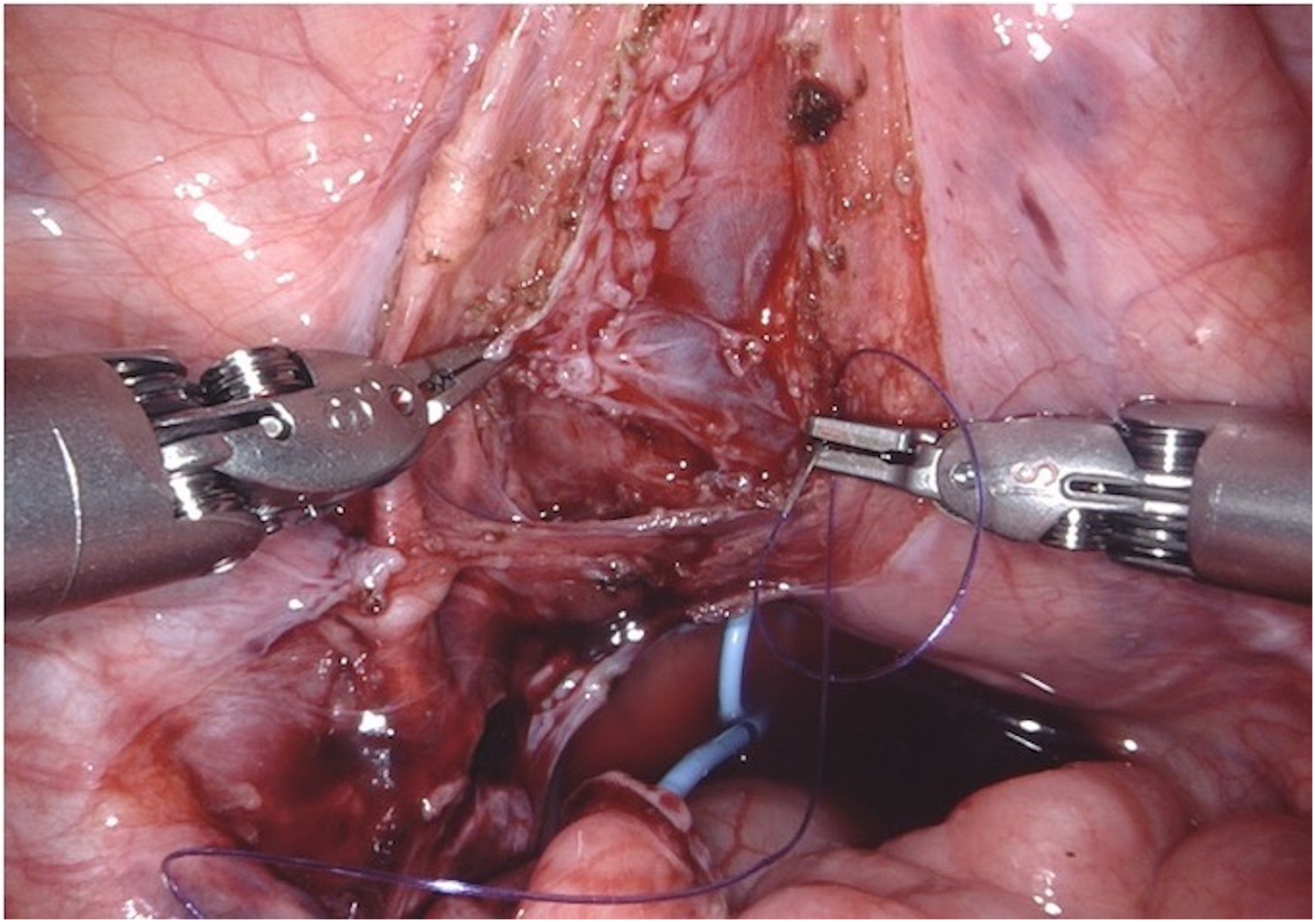
D-RALUR for OM. The detrusor muscle is incised sagittally in an inverted “Y” shape until the bladder mucosa is exposed. Care is taken to ensure that the ureterovesical junction and detrusotomy are aligned in a straight line to prevent angulation. The resulting submucosal tunnel measures 4–5 cm in length.

**Figure 4:**
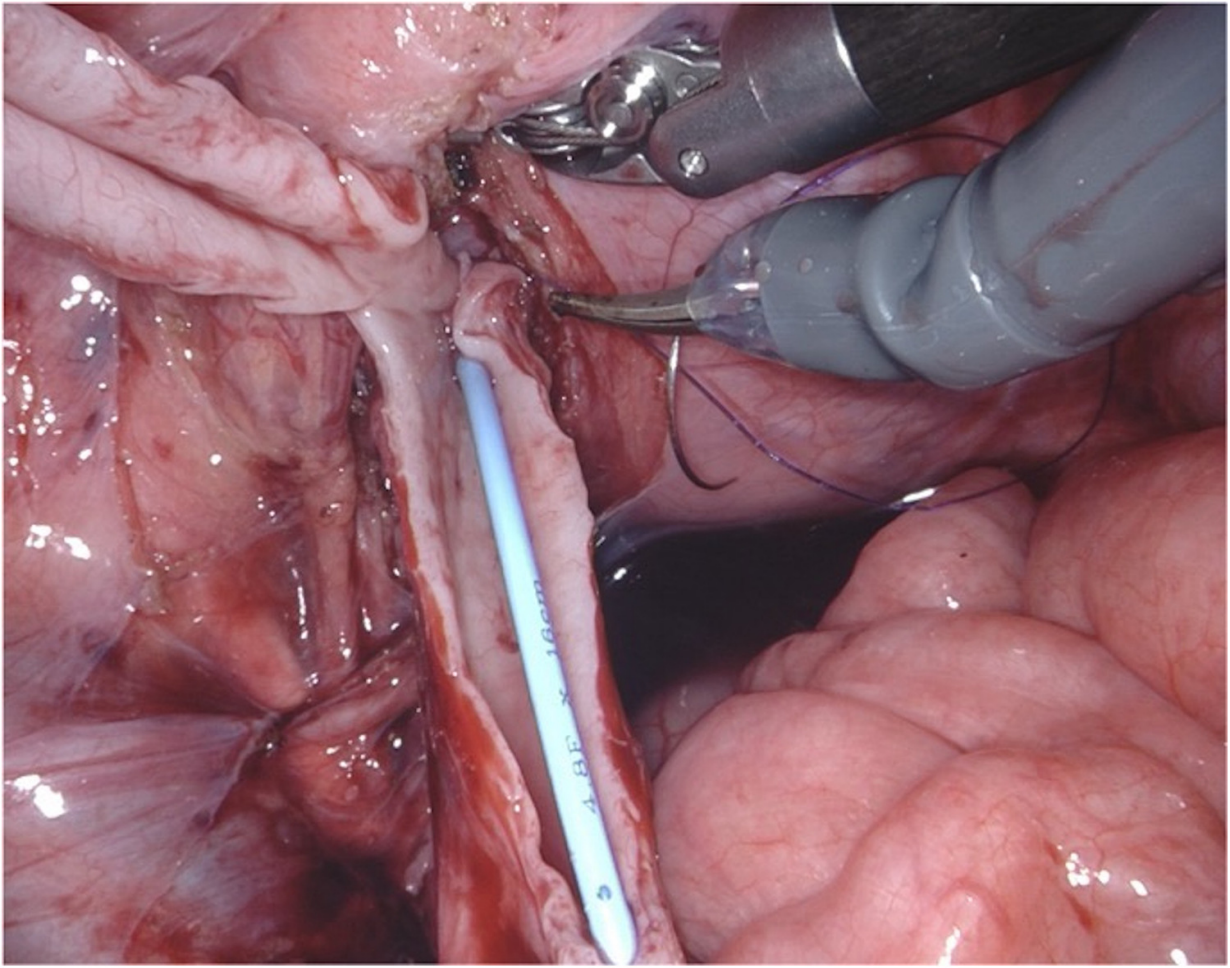
D-RALUR for OM. Ureteral tapering is performed using the Hendren technique: redundant ureter is excised over a JJ stent.

**Figure 5:**
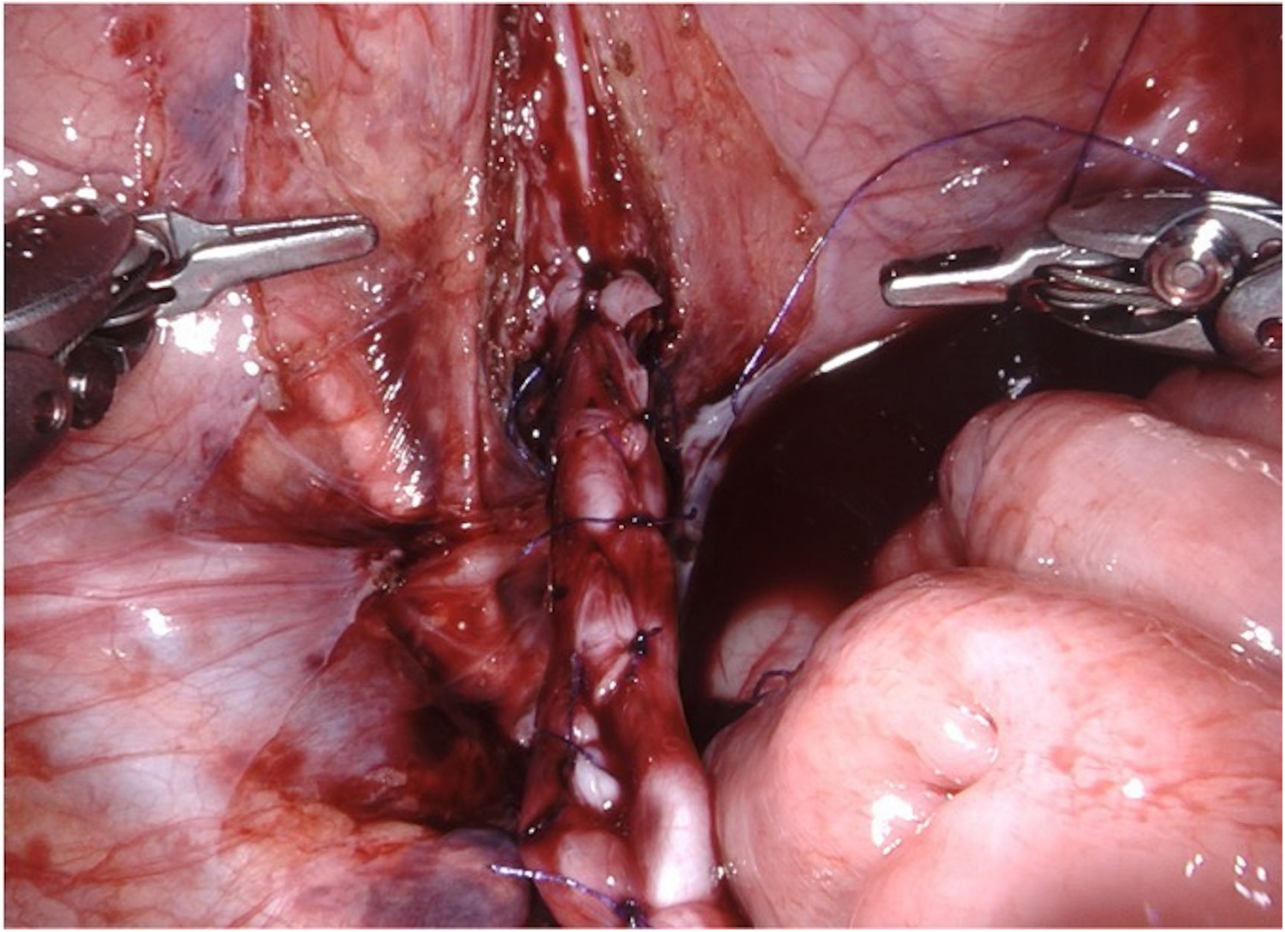
D-RALUR for OM. Ureteral tapering is performed using the Hendren technique: the ureter is sutured by interrupted two layers sutures.

**Figure 6:**
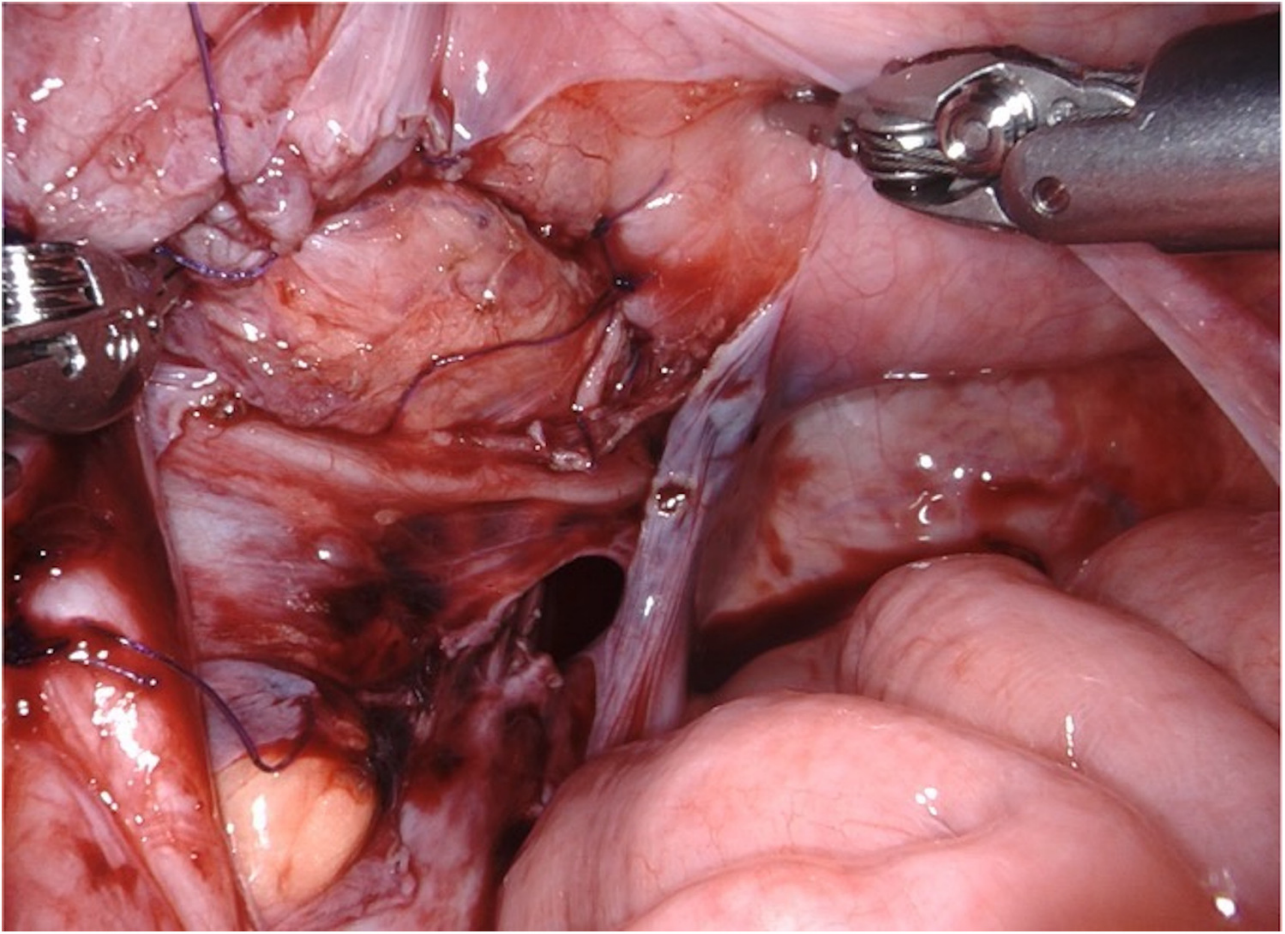
D-RALUR for OM. The detrusor muscle is wrapped around the ureter with interrupted sutures, and the ureteral adventitia is included in each stitch.

LUR and RALUR were performed using the Lich-Gregoir technique (20), as initially described by Ehrlich et al. (5) for LUR and by Peters et al. (21) for RALUR. RALUR followed the LUAA recommendations, as outlined by Gundeti et al. (22). The patient was placed in a supine position with a 20° Trendelenburg tilt, and a Foley catheter was inserted intraoperatively. In LUR, a 12 mm camera port was introduced through the umbilicus using the Hasson technique, and pneumoperitoneum was established with CO2 insufflation to a pressure of 10 mmHg. Two additional 5 mm working ports were placed under direct visualization along the transverse umbilical line, one on the right and one on the left. For RALUR, the Da Vinci Surgical Robot Xi® system was used. An 8 mm camera port with a 0° lens was inserted through the umbilicus using the Hasson technique, and pneumoperitoneum was created by insufflating CO2 to a pressure of 10 mmHg. Two to three additional 8 mm working ports (depending on the patient's size) were placed under direct visualization along a transverse line intersecting the umbilicus, ensuring a minimum separation of approximately 4 cm between each port. The robot was then docked. The distal ureter was identified and mobilized caudally, crossing the iliac vessels toward the UVJ, while preserving the ductus deferens or uterine arteries. A tape around the ureter may be used for atraumatic handling. The bladder was filled with sterile saline, and a transabdominal stitch could be used to elevate the bladder for improved visualization. A sagittal incision in an inverted “Y” shape was made in the detrusor muscle until the mucosal layer was visible, ensuring the UVJ and detrusotomy were aligned in a straight line to prevent angulation. The created tunnel measured 4–5 cm in length. In dismembered procedures, the distal portion of the ureter was excised from the UVJ as distally as possible, and the terminal part was removed. A JJ stent was introduced through one of the robotic trocars or a 3 mm assistant port. The neo-ureteral opening was created near the native meatus (if in orthotopic position). In males, the ureter, which crosses the vas deferens in this region, was repositioned above this structure, and the ureterovesical anastomosis was performed using interrupted 5-0 polydioxanone stitches. The ureter was then placed within the detrusor tunnel. A U stitch was placed at the distal end of the detrusotomy to advance the ureter by suturing the detrusor at the 5 o'clock position, followed by the ureteral adventitia, and then the detrusor at the 7 o'clock position. A 5-0 polydioxanone permanent stitch was placed at the apex of the detrusotomy through the ureteral adventitia to align the ureter within the tunnel and prevent slippage after surgery. Detrusorrhaphy was performed with interrupted stitches starting at the distal aspect of the detrusotomy, incorporating the ureteral adventitia. In cases of DS, both ureters were inserted into a wider, shared submucosal tunnel. If the ureter was dilated, an intra-corporeal tailoring or tapering procedure was performed in dismembered cases. The bladder was then filled with saline to check for urinary leakage or ureteral kinking. In cases of a short ureter, ureteral kinking, or excessive tension on the anastomosis, mobilizing the anterior bladder wall and performing a psoas hitch may be helpful to reduce stress on the ureter (23, 24). A perivesical drain was placed when necessary. Figures 1–6 illustrate D-RALUR performed for OM.

## 3 Results

A total of 162 patients underwent ureteral reimplantation during the study period: 61 OUR, 21 LUR (16 ND-LUR and 5 D-LUR), and 80 RALUR (18 ND-RALUR and 62 D-RALUR). Demographics and diagnostic characteristics are summarized in Table 1. Twenty-seven patients (17%) had DS (15 OUR, 2 LUR, and 10 RALUR).

**Table 1:** Patient demographics and diagnosis characteristics.

Characteristic	OUR	LUR	RALUR	RALUR vs. OUR	RALUR vs. LUR
	*n* = 61	*n* = 21	*n* = 80	*p*	*p*
	*n* (%) or median (IQR)	*n* (%) or median (IQR)	*n* (%) or median (IQR)		
Male	40 (66%)	17 (81%)	57 (71%)	0.942	0.742
Age (years)	2.1 (1.01–3.7)	1.7 (1.0–3.1)	3.3 (1.8–6.0)	0.005	0.013
Weight (kg)	12.0 (9.8–16.0)	13.5 (11.0–15.0)	15.0 (12.0–20.0)	0.003	0.242
Antenatal diagnosis	37 (61%)	8 (38%)	37 (46%)	0.180	0.999
Diagnosis				n.a.	n.a.
VUR	31 (51%)	16 (76%)	40 (50%)		
OM	22 (36%)	3 (14%)	28 (35%)		
ROM	8 (13%)	2 (10%)	12 (15%)		
Mean VUR grade^a^				0.999	0.564
2	2 (5%)	0 (0%)	3 (6%)		
3	4 (10%)	4 (22%)	6 (11%)		
4	23 (59%)	5 (28%)	25 (47%)		
5	10 (26%)	9 (50%)	19 (36%)		
Duplex system	15 (25%)	2 (10%)	10 (13%)	0.126	0.999
Paraureteric vesical diverticulum	11 (18%)	2 (10%)	16 (20%)	0.999	0.700
Ureterocele	10 (16%)	0 (0%)	6 (8%)	0.198	0.680
Previous surgical UVJ treatment	23 (38%)	11 (52%)	46 (56%)	0.058	0.999
Pre-operative functional study performed					
Renal scintigraphy	16 (26%)	5 (24%)	17 (21%)	0.978	0.999
fMRU	21 (34%)	10 (48%)	59 (74%)	< 0.001	0.044

OUR, open ureteral reimplantation; LUR, laparoscopic ureteral reimplantation; RALUR, robot-assisted ureteral reimplantation; VUR, vesico-ureteral reflux; OM, obstructive megaureter; ROM, refluxing obstructive megaureter; fMRU, functional magnetic resonance urography; UVJ, ureteral-vesical junction; OM, obstructive megaureter; IQR, interquartile range; p, Bonferroni's adjusted *p*-value. n.a., not applicable.

^a^ Percentages were calculated excluding the 52 patients without VUR.

Surgical and early postoperative data are summarized in Table 2. In RALUR, three trocars were required in 18 patients (22%), and four trocars were required in 62 patients (78%), placed with a mean distance of 4.6 cm (range 4–7, SD 0.5). The mean detrusor tunnel length was 4.7 cm (range 2–6, SD 0.7). Ureteral remodelling was performed in 55 cases: 38 Hendren tapering (24 OUR, 14 RALUR), 2 Kalicinski (1 OUR, 1 RALUR), and 15 Starr tailoring (7 OUR, 8 RALUR). Ureteral stent cases requiring dismembering the ureter had a mean stent duration of 27.9 days in OUR (range 4–90, SD 18.7), 36 days in D-LUR (range 19–55, SD 13), and 51 days in D-RALUR (range 3–191, SD 26). Abdominal drainage was more frequently placed in OUR than in RALUR (32 vs. 16 patients, *p* < 0.001), and bladder catheter duration was significantly longer in OUR than in RALUR (median values: 4 days vs. 1 day, *p* < 0.001). No postoperative urinary retention was detected after catheter removal, even following bilateral repair. Additionally, hospital stay was shorter after RALUR than after OUR (*p* < 0.001), and patients undergoing RALUR required fewer and shorter courses of analgesic medications. No intraoperative complications or conversions to open surgery were reported for laparoscopic and robotic procedures. Success rates, postoperative findings, and complication management are reported in Table 3. Five patients (3 OUR, 1 LUR, and 1 RALUR) were lost to follow-up and were not considered in the outcome evaluation. Success rates were 79%, 50%, and 65% for OUR, LUR, and RALUR, respectively. Obstruction occurred in 3 cases in the OUR group (all treated by redo reimplantation), 1 case in the LUR group (treated by ureteral stenting), and 10 cases in the RALUR group. Among the patients with obstruction in the RALUR group, 6 cases (60%) were stented, and 4 cases (40%) had surgical procedures: 2 redo RALUR, 1 crossed robotic ureteroureterostomy, and 1 temporary ureteral diversion. Postoperative VUR was reported in 9 cases in the OUR group (15%), 9 cases in the LUR group (45%), and 18 cases in the RALUR group (22%). Twenty-four patients experienced persistent postoperative VUR. Among them, 4/24 had preoperative grade III VUR and developed postoperative grade III VUR. Of the 11/24 patients with preoperative grade IV VUR, 5/11 developed postoperative grade III VUR, while 6/11 had persistent grade IV VUR. Among the 9/24 patients with preoperative grade V VUR, 3/9 developed postoperative grade III VUR, 4/9 developed postoperative grade IV VUR, and 2/9 had persistent grade V VUR.

**Table 2:** Surgical and postoperative details.

Surgical and post-operative details	OUR	LUR	RALUR	RALUR vs. OUR	RALUR vs. LUR
	*n* = 61	*n* = 21	*n* = 80	*p*	*p*
	*n* (%) or median (IQR)	*n* (%) or median (IQR)	*n* (%) or median (IQR)		
Side				0.030	0.002
Right	16 (26%)	1 (5%)	30 (38%)		
Left	22 (36%)	19 (90%)	37 (46%)		
Bilateral	23 (38%)	1 (5%)	13 (16%)		
Operative time (min)	110 (90–150)	75 (65–100)	135 (115–175)	0.001	<0.001
Drainage					
Patients (*n*)	32 (52%)	1 (5%)	16 (20%)	<0.001	0.230
Permanence (days)	6.0 (4.5–8.5)	4.0 (n.a.)	3.5 (2.0–5.5)	0.004	0.999
Bladder catheter					
Patients (*n*)	61 (100%)	21 (100%)	80 (100%)	n.a.	n.a.
Permanence (days)	4.0 (3.0–7.0)	1.0 (1.0–3.0)	1.0 (1.0–2.0)	<0.001	0.999
Hospital stay (days)	6.0 (5.0–10.0)	3.0 (2.0–4.0)	3.0 (2.0–4.0)	<0.001	0.999
Opioids treatment					
Patients (*n*)	38 (62%)	5 (24%)	9 (11%)	<0.001	0.322
Therapy days	3.0 (2.0–4.0)	1.0 (1.0–2.0)	1.0 (1.0–1.0)	<0.001	0.613
NSAIDs treatment					
Patients (*n*)	28 (46%)	13 (62%)	62 (78%)	<0.001	0.292
Therapy days	3.0 (2.0–3.0)	1.0 (1.0–1.0)	1.0 (1.0–2.0)	<0.001	0.134
Paracetamol treatment					
Patients (*n*)	61 (100%)	21 (100%)	80 (100%)	n.a.	n.a.
Therapy Days	5.0 (4.0–7.0)	3.0 (2.0–3.0)	2.0 (2.0–3.0)	<0.001	0.525

OUR, open ureteral reimplantation; LUR, laparoscopic ureteral reimplantation; RALUR, robot-assisted ureteral reimplantation; NSAIDs, non-steroidal anti-inflammatory drugs; IQR, interquartile range; *p*, Bonferroni's adjusted *p*-value. n.a., not applicable.

**Table 3:** Success rate and post-operative findings.

Post-operative complications or recurrence and success rate^a^	OUR	LUR	RALUR	RALUR vs. OUR	RALUR vs. LUR
	*n* = 58	*n* = 20	*n* = 79	*p*	*p*
	*n* (%) or median (IQR)	*n* (%) or median (IQR)	*n* (%) or median (IQR)		
Success rate	46 (79%)	10 (50%)	51 (65%)	0.122	0.464
Persistent VUR (*n*)					
Patients (*n*)	6 (10%)	5 (25%)	13 (16%)	0.614	0.752
Grade				0.999	0.980
3	3 (50%)	2 (40%)	7 (54%)		
4	2 (33%)	2 (40%)	6 (46%)		
5	1 (16.7%)	1 (20%)	0 (0%)		
Secondary VUR (*n*)					
Patients (*n*)	3 (5%)	4 (20%)	5 (6%)	0.999	0.156
Grade				0.999	0.999
3	1 (33%)	0 (0%)	1 (20%)		
4	2 (67%)	4 (100%)	3 (60%)		
5	0 (0%)	0 (0%)	1 (20%)		
Obstruction (*n*)	3 (5%)	1 (5%)	10 (13%)	0.280	0.908
Re-intervention					
Patients (*n*)	12 (21%)	10 (50%)	28 (36%)	0.108	0.496
Type				0.162	0.999
STING	6 (50%)	7 (70%)	16 (57%)		
Stent	0 (0%)	1 (10%)	6 (21%)		
Surgical redo	6 (50%)	2 (20%)	6 (21%)		
Resolution after re-intervention	10 (83%)	9 (90%)	26 (93%)	0.999	0.999
Endoscopic	5 (83%)	7 (88%)	22 (100%)	0.428	0.534
Surgical	5 (83%)	2 (100%)	4 (67%)	0.999	0.999
Overall success rate	56 (97%)	19 (95%)	77 (98%)	0.999	0.992
Follow up time (months)	45.0 (31.6–59.8)	21.5 (13.5–32.5)	14.0 (9.0–21.0)	<0.001	0.071

OUR, open ureteral reimplantation; LUR, laparoscopic ureteral reimplantation; RALUR, robot-assisted ureteral reimplantation; VUR, vesico-ureteral reflux; STING, subureteric transurethral injection; IQR, interquartile range; *p*, Bonferroni's adjusted *p*-value.

^a^ 5 Patients were excluded due to the lack of information after the surgical intervention.

VUR was managed by endoscopic bulking agent injection in 6 (50%), 7 (70%), and 16 (57%) patients, respectively; redo ureteral reimplantation was performed in the remaining cases. Surgical or endoscopic reintervention was successful in 10/12 cases in the OUR group, 9/10 cases in the LUR group, and 26/28 cases in the RALUR group.

Success rates, postoperative findings, and management of complications for ND-RALUR and D-RALUR are reported in Table 4. No statistically significant difference in outcome was observed between the two groups. Comparing ND-RALUR and D-RALUR, the ND-RALUR group had a higher number of postoperative obstructions (*p* = 0.005), while the D-RALUR group developed more postoperative VUR (*p* = 0.009).

**Table 4:** Success rate and post-operative findings in ND-RALUR and D-RALUR.

Post-operative complications or recurrence and success rate in ND and D-RALUR	ND-RALUR	D-RALUR	*p*
	*n* = 17	*n* = 62	
	*n* (%)	*n* (%)	
Success rate	11 (65%)	40 (65%)	0.988
Secondary/persistent VUR (*n*)	0 (0%)	18 (29%)	0.009
Obstruction (*n*)	6 (35%)	4 (6%)	0.005
Re-intervention			
Patients (*n*)	6 (35%)	22 (35%)	0.953
Type			<0.001
STING	0 (0%)	16 (73%)	
Stent	5 (83%)	1 (5%)	
Surgical redo	1 (17%)	5 (23%)	
Resolution after endoscopic re-intervention	5 (100%)	17 (100%)	n.a.
Resolution after surgical re-intervention	1 (100%)	3 (60%)	0.999
Overall success rates	17 (100%)	60 (97%)	0.999

ND-RALUR, non-dismembered robot-assisted ureteral reimplantation; D-RALUR, dismembered robot-assisted ureteral reimplantation; STING, subureteric transurethral injection; n.a., not applicable.

Success rates, postoperative findings, and complication management for RALUR performed in 2020–2021 and those performed in 2022–2023 are presented in Table 5. The postoperative success rate was 55% for the 51 RALUR procedures performed in 2020%–2021% and 81% for the 27 RALUR procedures performed in 2022–2023 (*p* = 0.020), resulting in endoscopic or surgical reintervention in 45% of patients in the first group and 18% of patients in the second group (*p* = 0.026). No statistically significant differences were reported for the overall success rate after endoscopic or surgical reinterventions between the two groups.

**Table 5:** Success rate and post-operative findings in RALUR 2020–2021 and RALUR 2022–2023.

Post-operative failures or recurrence and success rate in RALUR 2020–2021 vs. RALUR 2022–2023	RALUR	RALUR	*p*
	2020–2021	2022–2023	
	*n* = 51	*n* = 27	
	*n* (%)	*n* (%)	
Success rate	28 (55%)	22 (81%)	0.020
Secondary/persistent VUR (*n*)	15 (29%)	3 (11%)	0.092
Obstruction (*n*)	8 (16%)	2 (7%)	0.480
Re-intervention			
Patients (*n*)	23 (45%)	5 (18%)	0.026
Type			0.999
STING	13 (57%)	3 (60%)	
Stent	5 (21.7%)	1 (20%)	
Surgical redo	5 (21.7%)	1 (20%)	
Resolution after endoscopic re-intervention	18 (100%)	4 (100%)	n.a.
Resolution after surgical re-intervention	3 (60%)	1 (100%)	n.a.
Overall success rate	49 (96%)	27 (100%)	0.541

RALUR, non-dismembered robot-assisted ureteral reimplantation; STING, subureteric transurethral injection; n.a., not applicable

## 4 Discussion

The most commonly used technique for treating VUR is the endoscopic injection of bulking agents. When this is not indicated or proves ineffective, OUR is recommended. For other UVJ pathologies, such as OM, endoscopic treatments like stent placement, high-pressure balloon dilation (HPBD), and incision have been described. However, the long-term outcomes of these approaches remain uncertain. Consequently, the EAU Paediatric Urology Guidelines Panel does not recommend routine endoscopic management due to unclear intervention strategies and outcomes, making OUR the preferred approach (2, 4, 25). OUR can be performed using either an intravesical or extravesical approach (4). Among open techniques, the most widely used and reliable vesicoureteral reimplantation method is the cross-trigonal procedure described by Cohen, which has an excellent success rate (9, 21, 26). However, its main drawback is the alteration of the ureteral orientation, which may complicate future endoscopic access to the ureters, if needed. Additionally, OUR is associated with significant postoperative pain, hematuria, dysuria, and prolonged hospitalization (27). Minimally invasive surgery (MIS) for VUR treatment is undoubtedly more invasive than endoscopic treatment, and its advantages over open surgery have not yet been clearly established. As a result, MIS cannot currently be considered a first-line approach. Nevertheless, it may be proposed as an alternative in high-volume centers with proven expertise in MIS or in cases of failure or complex anatomy, particularly robotic surgery, as demonstrated by Esposito et al. in their recent study (4, 28). LUR for the treatment of VUR and OM has been described with a reported success rate ranging from 87%–100% (27, 29–31). Despite its feasibility, this procedure has not been widely adopted due to the advanced laparoscopic skills required and limited ergonomics, particularly in bilateral or dismembered procedures (9, 15, 22, 27). MIS has regained attention with the advent of robotic surgery, which significantly simplifies the technical execution of this procedure thanks to its well-recognized advantages. Furthermore, robotic surgery offers a shorter and more accessible learning curve compared to conventional laparoscopy (27). Regarding D-RALUR for the treatment of megaureter and other UVJ anomalies, the available literature is still limited, reporting only a few case series. Neheman and Rappaport have published two studies describing the technical aspects of robot-assisted laparoscopic ureteral reimplantation for the management of OM with a novel approach. They compared robot-assisted cross-trigonal extravesical dismembered vesicoureteral reimplantation (RADECUR) and laparoscopic cross-trigonal extravesical dismembered ureteral reimplantation (LDECUR), respectively (32, 33). The RADECUR technique follows the ureteral orientation of the Cohen intravesical technique but with an extravesical approach. While the initial results are encouraging, it still has the disadvantage of altering the normal anatomy of the ureters, potentially hindering future endoscopic procedures.

Preserving normal UVJ anatomy and ureteral alignment in LUR and RALUR is particularly crucial for patients with congenital anomalies of the kidney and urinary tract (CAKUT), who may develop urinary stones over their lifetime and require endoscopic interventions. Furthermore, maintaining the normal alignment of the UVJ and ureter also allows for endoscopic approaches to complicated cases involving obstruction or VUR, thus avoiding the need for redo surgeries. As shown by our results, most re-interventions in the OUR group were treated with surgical re-intervention, while in the LUR and RALUR groups they were treated with endoscopic re-intervention. In the OUR group, endoscopic management of complications such as VUR and obstruction was feasible in only 6 out of 12 complicated cases (50%), while in the MIS cases, endoscopic treatment was possible in 30 out of 38 complicated patients (79%). Avoiding repeated surgeries on the UVJ is critical to prevent injury to the deferent duct and neurovascular bundle and to maintain the vascularization of the ureter, which could otherwise lead to recurrent stenosis.

Regarding postoperative complications, *de novo* or secondary VUR and obstruction were reported in our series. In the subgroup analysis of D-RALUR and ND-RALUR, we noticed that despite the inherently higher risk of urine leakage in D-RALUR, none of the patients in our cohort experienced this issue, as also reported in the study by Mittal (15). Interestingly, there was a significant difference in the type of postoperative failure: secondary or persistent VUR in the D-RALUR group (*p* = 0.009), and obstruction in the ND-RALUR group (*p* = 0.005). This result invites reflection on possible measures to reduce the incidence of these complications. In our experience with ND-RALUR and ND-LUR, obstruction has often been associated with ureteral kinking just outside the detrusor sleeve or with overly tight suturing of the detrusor. Therefore, we believe it is essential to ensure that the detrusor suture is not excessively tight, and that the ureter does not experience excessive tension or kinking outside the detrusor tunnel. Performing proximal mobilization of the ureter or mobilizing the bladder and executing a psoas hitch may help reduce the incidence of obstruction.

We did not report any intraoperative complications or need for conversion to open surgery, confirming that LUR and RALUR are safe and feasible procedures in the pediatric population, even in bilateral cases. Transient urinary retention is a well-known complication reported in cases of bilateral extravesical reimplantation, with a reported incidence in the literature ranging from 0%–37.5% (34). Neuroanatomic studies by Leissner and Yucel found that the main part of the pelvic plexus is approximately 1.5 cm dorsal and medial to the UVJ, with nerves present on the medial aspect of the distal ureter and encircling the ureter at the UVJ (35, 36). Based on these findings, it has been suggested that limiting distal dissection at the dorsomedial portion of the ureter and UVJ can reduce the risk of transient urinary retention (37). Moreover, urinary retention appears to occur more frequently in patients with bladder and bowel dysfunction (BBD) (13), which is considered the primary risk factor. In our series, we treated 13 bilateral cases (6 ND-RALUR and 7 D-RALUR) and did not observe any cases of postoperative urinary retention. We believe that, as suggested by Hajiyev and Gundeti (22), limiting the dissection over the distal 1.5 cm of the ureter, staying close to the ureteral adventitia, and avoiding cautery use may help minimize the risk of neurovascular bundle injury. In our series, the bladder catheter was removed earlier in patients from the LUR and RALUR cohorts compared to those in the OUR group. Additionally, LUR and RALUR showed statistically significant advantages over OUR, including reduced need for abdominal drainage, decreased analgesia requirements, and shorter hospital stays, further supporting the well-known benefits of MIS in fast-track surgery. However, operative times were longer in the RALUR group compared to OUR and LUR. LUR had the shortest operative times, with a statistically significant difference compared to RALUR. This may be attributed to the additional time required to set up the robot. Moreover, our series included a higher number of dismembered procedures and ureteral remodeling cases in the RALUR group, contributing to the longer operative times. In LUR, ureteral remodeling can be performed using an intracorporeal technique (38), although cases of extracorporeal remodeling have also been described (39). Based on our experience, the enhanced dexterity provided by robotic surgery allows for easier intracorporeal ureteral remodeling compared to laparoscopy.

In our series, OUR, LUR, and RALUR did not show any statistically significant differences in success and reoperation rates. However, our results do not align with the success rates reported in the literature, even for OUR (9, 27, 30). In this study, LUR was performed on a small number of cases and for a limited period. Given the technical challenges and slower learning curve associated with LUR, this may explain the lower success rates observed. Once the robot became available at our institute, LUR was abandoned, and we focused on the development of RALUR. Even in this group, the results of the initial experience did not align with those reported in the literature (37), although they showed better results compared to LUR, even if not statistically significant. The learning curve played a significant role in our initial RALUR outcomes. When comparing cases treated in the first two years with those in the subsequent two years, the success rate increased from 55%–81%, approaching the rates reported in the literature and those observed in our OUR series. This suggests that increasing surgeon experience and the implementation of technical improvements positively impact the success rate of this technique. A similar trend was reported by Boysen et al. In fact, in their 2017 study, involving 260 children who underwent robotic ureteral reimplantation between 2005 and 2014, the success rate was 87.9%, and the complication rate was 2.7%. Subsequently, in 2018, among 143 patients with primary VUR treated with RALUR between 2015 and 2017, the success rate exceeded 93.8%, and the complication rate was 2.5% (11, 40). Another example of overcoming the learning curve is provided by Sahadev et al. (41). In their study, they describe several minor technical modifications implemented during 170 RALUR procedures that contributed to achieving acceptable outcomes while reducing morbidity. Some of these align with the technical suggestions described by Gundeti et al. (22), which we also adopted in our experience with RALUR and believe may have played a role in the improvements observed when comparing our first two-year experience with the subsequent two-year experience with RALUR.

In our series, with increased surgeon experience and refinements in technique, RALUR achieves success rates comparable to those of OUR while offering additional benefits such as shorter hospitalization, enhanced visualization, a lower risk of injury to the vas deferens and neurovascular structures, and improved access to the ureter for potential future endoscopic procedures. This study has several limitations. OUR and LUR patients were enrolled retrospectively, while RALUR patients prospectively, leading to potential biases in follow-up duration (longer for OUR and LUR) and selection, as these procedures ceased at our center after adopting robotic-assisted surgery. The surgical techniques compared were still being refined, especially dismembered procedures, and there was an early shift from laparoscopic surgery to robotic approaches. Various procedures and surgical indications were included. Additionally, OUR was performed by different surgeons rather than by the same surgeon as with LUR and RALUR. This factor may have introduced heterogeneity into the results and may be associated with the low success rate of OUR and the reported complications, as it reflects the varying surgical skills of multiple surgeons, despite all being experienced. In contrast, LUR and RALUR outcomes reflect the expertise of a single surgeon. Furthermore, a postoperative VCUG was not routinely performed in all patients but only in cases of recurrent urinary tract infections after surgery, leaving the true radiographic success rate unknown. Our results with RALUR are not yet optimal and reflect the initial learning curve. However, between the first and last two years of experience, RALUR achieves a success rate comparable to that reported in the literature and in our series for OUR. Additionally, it offers shorter hospitalization, improved visualization with a lower risk of injury to the vas deferens and vascular-nervous structures, no episodes of bladder paralysis or spasms, and easier access to the ureter for potential future endoscopic procedures through the extravesical approach. These undeniable advantages, combined with a simpler learning curve compared to laparoscopy, encourage robotic ureteral reimplantation for VUR treatment and other UVJ pathologies using the D-RALUR technique. Nevertheless, the ideal candidates for this approach remain a topic of discussion. In conclusion, the continued use of robotic ureteral reimplantation is warranted due to its promising outcomes, which improve with surgeon experience, as well as its advantages in clinical practice. Further studies with larger, more homogeneous cohorts and greater surgical experience are needed to validate these preliminary outcomes and refine surgical techniques.

## Data Availability

The original contributions presented in the study are included in the article/Supplementary Material, further inquiries can be directed to the corresponding author.
