# Supplementary material for: From open to robotic surgery in pediatric ureteral reimplantation: overcoming the learning curve for improved outcomes
Source: Front Surg. 2025 Apr 29;12:1573233. doi: 10.3389/fsurg.2025.1573233 (PMC12069337; doi:10.3389/fsurg.2025.1573233)
Supplement: Supplementary file 1 [file Table1.docx]

Supplementary Material

# Supplementary Table

**Supplementary Table 1.** Post-operative success rate and complications. VUR = Vesico-Ureteral Reflux; UVJO = Uretero-Vesical Junction Obstruction; OUR = Open Ureteral Reimplantation; LUR = Laparoscopic Ureteral Reimplantation; RALUR = Robot-Assisted Ureteral Reimplantation; *162 is the total number of patients enrolled in the study, but 5 patients (3 OUR, 1 LUR, and 1 RALUR) were lost to follow-up and were not included in the outcome evaluation

| **Post-operative**  **success rate and complications*** | **Success rate**  **n (%)** | **Complications**  **n (%)** | **Follow up (months)**  **mean (range)** |
| --- | --- | --- | --- |
| **Unilateral VUR**  n = 56/157* (36%)  **OUR n =** 12/56 (21%)  **LUR n =** 15/56 (27%)  **RALUR n =** 29/56 (52%) | 39/56 (70%)  10/12 (83%)  9/15 (60%)  20/29 (69%) | 17/56 (30%)  2/12 (17%)  6/15 (40%)  9/29 (31%) | 25.5 (6-69.7) |
| **Bilateral VUR**  n **=** 28/157* (18%)  **OUR** n = 18/28 (64%)  **LUR** n = 0  **RALUR** n = 10/28 (36%) | 22/28 (79%)  16/18 (89%)  0  6/10 (60%) | 6/28 (21%)  2/18 (11%)  0  4/10 (40%) | 36.6 (9-79.7) |
| **Unilateral UVJO**  n = 68/157* (43%)  **OUR** n = 25/68 (37%)  **LUR** n = 5/68 (7%)  **RALUR** n = 38/68 (56%) | 45/68 (66%)  19/25 (76%)  1/5 (20%)  25/38 (66%) | 23/68 (34%)  6/25 (24%)  4/5 (80%)  13/38 (34%) | 26.9 (6-102.3) |
| **Bilateral UVJO**  n = 5/157* (3%)  **OUR** n = 3/5 (60%)  **LUR** n = 0  **RALUR** n = 2/5 (40%) | 1/5 (20%)  1/3 (33.3%)  0  0/2 | 4/5 (80%)  2/3 (66.7%)  0  2/2 (100%) | 25.9 (9-36.4) |
| **VUR associated with complex anatomy or prior surgical UVJ treatment**  n = 69/157* (44%)  **OUR** n = 25/69 (36%)  **LUR** n = 10/69 (14)  **RALUR** n = 34/69 (50%) | 51/69 (74%)  22/25 (88%)  6/10 (60%)  23/34 (68%) | 18/69 (26%)  3/25 (12%)  4/10 (40%)  11/34 (32%) | 29.5 (6-79.7) |
